# Adverse Changes in Body Composition During the Menopausal Transition and Relation to Cardiovascular Risk: A Contemporary Review

**DOI:** 10.1089/whr.2021.0119

**Published:** 2022-06-13

**Authors:** Varna Kodoth, Samantha Scaccia, Brooke Aggarwal

**Affiliations:** ^1^ Division of Cardiology, Department of Medicine, Columbia University Irving Medical Center, New York, New York, USA.; ^2^ Institute of Human Nutrition, Columbia University Irving Medical Center, New York, New York, USA.

**Keywords:** menopausal transition, weight gain, body composition, aging, hormonal changes, cardiovascular disease

## Abstract

The menopausal transition period in aging women is strongly associated with weight gain. Evidence shows that weight changes during menopause increases the risk of developing cardiovascular disease (CVD) in postmenopausal women. However, the potential mechanisms that cause weight gain and adverse changes to body composition specifically during the menopausal transition period remain to be elucidated. In this contemporary review, we examined recent evidence for adverse changes in body composition at midlife during the menopausal transition and the link to increased CVD risk and described factors that may contribute to these changes, including normal chronological aging, hormonal factors (decreased estrogen, etc.), behavioral factors (changes in diet, physical activity), or other emerging factors (*e.g.*, sleep). This review focused on identifying factors that make the menopausal transition period a critical window for prevention of CVD. Future study is needed to decipher the extent to which hormonal changes, age-related factors, and behavioral factors interact with and contribute to increased CVD risk in women undergoing menopause. Understanding the causes of weight gain during the menopausal transition may help to inform strategies to mitigate adverse CVD outcomes for women transitioning through menopause.

## Introduction

Overweight and obesity are well-established risk factors for cardiovascular disease (CVD).^1^ Women are particularly prone to obesity during reproductive life stages such as pregnancy and menopause.^2^ Weight gain is a symptom of menopause experienced by 60%–70% of midlife women.^3^ On average, women gain about 1.5 pounds per year during the midlife period (age 50–60 years), independent of their initial body size or race/ethnicity.^2^ Age is strongly associated with involuntary loss of muscle mass such that after the age of 30, muscle mass declines by 3%–8%.^4^

The trajectory of changes in body composition for women undergoing menopause has recently been characterized and it entails a significant acceleration of fat mass increase and lean mass decline during the menopausal transition, and a stabilization of body composition postmenopause.^5^ Shifts in fat distribution and in body composition that occur during the menopausal transition are associated with increased cardiometabolic risk factors, including elevated blood pressure, elevated low-density lipoprotein (LDL), and obesity.^6^ Changes in visceral fat, in particular, increase CVD risk by associating with insulin resistance, inflammation, and adverse lipid profile.^7,8^

Traditional cardiovascular risk factors like obesity disproportionately impact midlife women. In an analysis of the National Health and Nutrition Examination Survey (NHANES) dataset, a nationally represented survey of adults in the United States, the prevalence of obesity in women (40.4%) is significantly higher than in men (35%) when adjusted for age-related factors.^9^ The effects of weight gain on CVD risk have been well studied; however, the causes of such weight gain during the menopausal transition are less understood.

Weight gain during the menopausal transition was previously thought to be a result of chronological aging alone. However, the Study of Women's Health across the Nation (SWAN), a longitudinal study of 3300 women, documented specific aspects of the transition that uniquely contributed to the development of obesity over and above age, including distinct patterns of hormone changes.^10^ Other studies have indicated that weight gain in midlife women may be primarily due to the hormonal changes associated with the menopausal transition.^2,6,11^

Similarly, dramatic increases in lipid measurements (total cholesterol, low-density lipoprotein-cholesterol [LDL-C], and apolipoprotein B levels) and metabolic syndrome (MetS) risk in women undergoing the menopausal transition have been shown to be independent of aging.^12^ Yet, whether these changes in body composition, fat distribution, and weight, are directly associated with the hormonal changes of the menopausal transition or simply due to aging, or other understudied factors (*e.g.*, behavioral changes) has been the subject of investigation in recent years.^2,13–16^ Despite the identification of the menopausal transition as a key period of detrimental changes in women's adiposity levels and overall CVD risk, the number of studies that are inclusive of women undergoing this transition are extremely limited.

The objective of this contemporary review is to review the body of evidence on factors influencing weight gain during the transition through menopause and resultant CVD risk factors in midlife women. First, we explore the differential impacts of menopausal weight gain on body fat composition and CVD risk in midlife women. Next, we review studies that describe the effects of hormones on adverse weight gain in women through an analysis of weight gain in premenopausal women in comparison to postmenopausal women. Third, we compare age-related effects on weight gain in women and the hormone-related effects on weight gain in women. A better understanding of the role of various factors involved in weight gain in women ages 40–55 years may be used to help inform menopausal weight gain prevention strategies and lifestyle practices to reduce CVD risk during this period.

## Definitions of Menopausal Stages

Menopause is defined as the last menstrual period followed by 12 or more months of absence of menstruation.^7^ Menopausal transition begins 5–10 years before the final menstrual period (FMP).^5^ Perimenopause refers to the first year that follows the FMP. This marks a period of increased variability in the duration and consistency of the menstrual cycle. The perimenopausal period is at the conclusion of a woman's reproductive years, which may begin as early as 40 or as late as 55.^2^ Postmenopausal refers to the period following a woman's FMP, during which the estrogen and follicle-stimulating hormone (FSH) levels stabilize.^17^

## Description of Adverse Weight Changes During Menopause

Perimenopause is marked by a significant decrease in estrogen hormone levels and the redistribution of subcutaneous fat to abdominal fat.^18^ Obesity and changes to body fat composition are closely associated with increased risk of CVD, particularly in postmenopausal women.^6,17^ In a Montreal–Ottawa New Emerging Team group study, 102 premenopausal women were followed for 5 years with annual measurements that included dual-energy X-ray absorptiometry (DXA).^6^ Women in the postmenopausal stage show a significant increase in visceral fat (*p* < 0.01) in comparison to their baseline when they were premenopausal.^6^ On average, visceral fat increases from 5%–8% of total body fat in the premenopausal state to 15%–20% of total body fat in the postmenopausal state.^2^ Through a variety of biomolecular mechanisms, visceral adipose tissue (VAT) is most strongly associated with increased cardiometabolic risk in obese women in comparison to other body fats.^19^

A recent study characterized the increase in VAT starting at 2 years before the onset of menopause as a key risk indicator of subclinical atherosclerosis in the internal carotid artery in 362 midlife women from Pittsburgh and Chicago who participated in the SWAN heart study.^20^ VAT increased significantly by 8.2% (95% CI: 4.1–12.5) and 5.8% (95% CI: 3.7–7.9) per year 2 years before FMP and after FMP.^20^ Samargandy et al. found that a 20% increase in VAT is associated with a 2% greater internal carotid artery.^20^ This suggests that the menopausal transition predisposes women to increased CVD risk independent of aging.

Many cross-sectional and longitudinal studies explore the relationship between the advancement of chronological and ovarian age through the menopausal transition and resultant adverse weight gain. Midlife obesity is frequently associated with age at the time of FMP.^2^ Early onset of menopausal transition was associated with increased body mass index (BMI), waist circumference, VAT, and subcutaneous adipose tissue (SAT) (*p* < 0.0001).^21^

### Menopausal weight gain and impact on CVD risk

The investigation of the relationship between menopause and CVD risk began as early as 1976 in The Framingham Study. In this study, from a cohort of 2873 women under the age of 55 years, the authors found the rate of CVD incidents to be lower in premenopausal women in comparison to postmenopausal women.^22^ Lissner et al. examined body weight variability within the Framingham Heart Study cohort. This study concluded that participants with highly variable body weights had increased total mortality (*p* = 0.005 for men, *p* = 0.01 for women), mortality from coronary heart disease (*p* = 0.009 for men, *p* = 0.009 for women), and morbidity due to coronary heart disease (*p* = 0.0009 for men, *p* = 0.006 for women).^23^ The analysis controlled for obesity, trends in weight over time, and indicators of cardiovascular risk, and found that the positive association with CVD risk and CVD mortality is attributable to weight changes.

Women gain 12 pounds within 8 years of the onset of menopause, on average.^24^ A longitudinal study by Gambacciani et al. found that an 8–20-pound increase in weight in women 34–59 years of age, increased risk of CVD by 27% (95% CI: 12–45) compared with women who maintained their baseline weight.^25^ Taken together, it is evident that weight gain attributed to the menopausal transition leads to increased CVD risk.

A cross-sectional study of 1422 middle-aged women from the Korean National Health and Nutrition Examination Survey (KNHANES), showed increased waist circumference in postmenopausal women to be strongly associated with increased systolic blood pressure, a known CVD risk factor.^26^ This study postulates that the significant changes in estrogen production are responsible for the changes in body fat distribution. Body fat composition was identified as a cause of the high prevalence of hypertension in postmenopausal women in comparison to premenopausal women (systolic blood pressure: 118.33 mm Hg, 95% CI: 116.52–120.15 vs. 115.22 mm Hg, 95% CI: 114.17–116.28, *p* = 0.003 and diastolic blood pressure: 76.94 mm Hg, 95% CI: 75.88–77.99 vs. 75.25 mm Hg, 95% CI: 74.57–75.93, *p* = 0.009, respectively) after adjustment for age.^26^ This study established the increase in blood pressure after menopause is related to the changes in body fat women experience during menopause.

Adverse changes in body composition persist across trunk, subcutaneous, and intra-abdominal fat deposits for postmenopausal women in comparison to premenopausal women. A cohort study seeking to elucidate the effect of menopausal transition on body fat distribution, studied 53 middle-aged premenopausal women and 28 early postmenopausal women over a 5-year period. This study measured upper body trunk fat, subcutaneous fat, and intra-abdominal fat concentrations through magnetic resonance imaging.^24^ From this, the investigators found that postmenopausal women gained 36% more trunk fat (*p* < 0.01), 49% greater intra-abdominal fat area (*p* < 0.01), and 22% greater subcutaneous abdominal fat area (*p* < 0.05) than premenopausal women.^24^ Thus, the change in body fat composition and distribution to the intra-abdominal area occurs specifically during menopausal transition and ultimately can lead to an increase in CVD risk factors such as hypertension, hyperlipidemia, and diabetes.

Several cross-sectional and longitudinal studies attribute changes to visceral fat deposition to transversion through menopausal states results in changes to visceral fat deposition. For example, in Gambacciani et al., the authors' conducted dual-energy X-rays on 8764 women in the authors' menopause clinic for body fat tissue distribution throughout menopausal transition. The major findings included a higher percentage of fat in the trunk region of perimenopausal and postmenopausal women in comparison to premenopausal women (*p* < 0.05).^25^ Additionally, the investigators found a higher percentage of mean total body fat and fat percentage relative to soft tissue in the body in perimenopausal and postmenopausal women in comparison to premenopausal women (*p* < 0.05).^25^ It is also noteworthy that this study showed that the shift in body composition is to a central fat distribution, with statistically insignificant changes to leg or arm fat deposits.

An observational and longitudinal study, with annual measurements for 4 years, assessed longitudinal changes in body fat composition across menopausal transition to identify an important distinction between VAT and SAT.^27^ Of the 156 women, premenopausal at the start of the study, 51 women became postmenopausal by the fourth year of the study. All postmenopausal participants gained a significant amount of VAT from baseline to year 4 (*p* < 0.05).^27^ The total area of SAT also increased; however, this increase is present across both perimenopausal and postmenopausal women at year 4 of the study, which suggests SAT weight gain occurs with aging and VAT weight gain occurs due to menopausal transition.^27^

The Women's Health Initiative is an investigation of disease prevention techniques to mitigate cardiovascular risk in postmenopausal women. From the Women's Health Initiative Observational Study cohort, 612 postmenopausal women aged 50–79 years were studied for the most relevant obesity-related modifiable and nonmodifiable risk factors over the course of 3 years.^28^ This study looked to target risk factors that caused a greater than or equal to 3% weight gain over the course of the study. Using a classification and regression tree (CART) analytic approach, the data showed that in postmenopausal women, the majority of the 3% weight gain is gained from the second annual study visit to the third annual study visit.^28^ Additionally, this study supported that body weight gain in postmenopausal women is associated with regaining previous weight loss (*p* < 0.01) in comparison to women not in the postmenopausal stage.^28^ This indicates that the menopausal transition not only leads to weight gain but also poses as a risk to the maintenance of weight loss.

To further examine weight gain following menopause another study followed-up on postmenopausal women that underwent a weight loss program. This program intervention involved 15 weeks of caloric restriction. This study included 19 obese postmenopausal women with no history of CVD. The women selected to be enrolled in this weight loss intervention gained 10.7 ± 5.6 kg during the postmenopausal period.^29^ After the intervention, the women reported significant declines in body weight in comparison to their body weight at baseline (*p* < 0.05).^29^ However, at the 12-month follow-up, women regained 2.5 ± 3.3 kg and exhibited increased BMI (*p* < 0.001), waist circumference (*p* < 0.001), and fat mass (*p* < 0.001), on average.^29^ This suggests that weight gain that occurs during menopausal transition may inhibit efforts to lose weight in the postmenopausal period and decrease risk of CVD in midlife women.

### Adverse weight gain due to hormonal factors

Women's rates of CVD increase exponentially during the postmenopausal stage as a result of weight gain associated with hormonal changes. During the early stages of menopausal transition, women experience estrogen deficiency.^30^ The reduction in estrogen leads to a decrease in lean body mass and an increase in fat mass.^28^ The Melbourne Women's Midlife Health Project is a 9-year-long prospective observational study with 438 native Australian women 45–55 years of age at the start of the study. The investigators found strong associations between CVD risk as a result of obesity and increased FSH levels and depressed estradiol (E2) levels during menopausal transition.

Estrogen is a regulator for adiposity.^31^ Specifically, estrogen promotes accumulation of subcutaneous fat.^31^ Following the onset of the menopausal transition, estrogen reduction instead leads to accumulation of central body fat deposits.^31^

In a prospective cohort study conducted by the University of Washington, 69 women were followed from the premenopausal stage to the postmenopausal stage.^32^ During this time, body composition was measured for changes in intra-abdominal fat. The results corroborate existing evidence that women experience weight gain during premenopausal status to postmenopausal status. Women presented with statistically significant adipokines and inflammatory markers associated with changes in abdominal fat.^32^ The data collected on intra-abdominal fat supported a proportionally greater increase in visceral adiposity and leads to an adverse metabolic profile in postmenopausal women. Additionally, the researchers collected data on adipokine levels in premenopausal and postmenopausal women that often serve as markers for obese individuals. The markers of interest included in this study were tissue plasminogen activator antigen (tPA), C-reactive protein (CRP), leptin, and adiponectin.

The findings of this study demonstrate that the increase in central adiposity or visceral adiposity over the course of menopausal transition was associated with adverse changes in leptin, tPA, CRP, and adiponectin. The change in intra-abdominal fat was positively correlated with increases in tPA (*p* < 0.005), CRP (*p* < 0.001), and leptin (*p* < 0.005).^33^ The increase in intra-abdominal fat was inversely related to adiponectin (*p* = 0.005) which is also consistent with the marker profile for obese subjects.^32^ At a hormonal level, it is evident that the changes in estradiol impact body fat composition. As numerous adipokines play a role in the development of CVD, it is important to highlight that the adipokines present in visceral fat may result in increased CVD risk in postmenopausal women.^32^

The role that changes in hormonal levels play influencing adverse body composition changes during menopausal transition in women was investigated in the SWAN, which documented patterns of hormone change for E2 and FSH. The SWAN study is a longitudinal observational cohort study of 3302 women ages 42–52 years, undergoing menopausal transition. In a recent longitudinal study using the SWAN cohort, researchers investigated the role of reproductive hormones on obesity incidence in relation to menopause status, independent of age-related factors. The results demonstrated that biologically available testosterone and sex hormone-binding globulin are independently predictive of incident obesity and severe obesity in menopausal women, regardless of age. When estradiol decreases drastically from perimenopause to postmenopausal, there is a shift toward androgen dominance in the hormonal profile.^34^

The study concluded that the hormonal shift from estradiol to higher free androgen index and lower sex hormone-binding globulin over the course of menopausal transition is strongly associated with increased risk of MetS. MetS is linked to the development of CVD in midlife women.^34^ These data suggest that hormonal changes during menopause promote subsequent obesity.

Consistent with these findings is the Third French MONICA cohort located in Toulouse area (South-western France), Lille area (North France), and Bas-Rhin area (East France). This cohort comprised 696 postmenopausal women, 183 perimenopausal women, and 659 premenopausal women. The study concluded that CHD risk in postmenopausal women is likely to be explained by estrogen deprivation.^35^ The investigators suggest that the observed changes within the body fat composition is attributable to the decrease in serum estrogen levels at the onset of menopause. Many studies show that estrogen induces increased uptake of LDLs.^35^ A lowered concentration of LDL is shown to protect women from developing atherosclerosis.^36^

Consistent with previous findings of the differential impacts to body fat composition in women, another study evaluated body weight and fat distribution in 2175 women in premenopausal, perimenopausal, and postmenopausal states and compared this cohort with 354 postmenopausal women undergoing forms of hormonal replacement therapy (HRT). The mean total body fat present in perimenopausal and postmenopausal women was higher than premenopausal women (*p* < 0.0001).^37^ This further supports the evidence that weight gain takes place in the intra-abdominal area over the course of menopausal transition. In the HRT-exposed postmenopausal group, the women exhibited body fat profile most similar to the premenopausal group not exposed to HRT. Taken together, it is clear that the hormonal changes play a significant role in fat accumulation over the course of menopausal transition.

### Adverse weight gain due to age-related factors

Existing studies support that aging and lifestyle determine weight gain during midlife independent of menopausal status.^1,2,5^ Since aging is associated with a decrease in lean muscle mass, resting metabolic rate, and total energy expenditure, it is possible that chronological aging, rather than hormonal changes, may be the primary influencer of weight status during this time.^2^

In a cross-sectional study on the effects of body composition changes in postmenopausal women, Wang et al. evaluated the contribution of aging versus menopause on body composition using DXA. Healthy postmenopausal women (*n* = 373) 50–60 years of age were recruited and then grouped based on age and years since menopause (YSM).^15^ Total body fat increased significantly with age (*r* = 0.12, *p* < 0.05) but not YSM. Furthermore, the amount of abdominal fat and abdominal fat percent increased with age (*r* = −0.15 to 0.16, *p* < 0.001) but not YSM. This was one of the first studies to show that total fat mass and change in fat distribution was associated with increasing age independent of YSM. Similarly, a study comparing body composition in 365 pre- and 201 postmenopausal Japanese women considered the contribution of aging and menopause to changes in regional lean and fat mass.^16^ Trunk fat mass and percentage of body fat were positively correlated with age (*p* < 0.001, *p* < 0.01 respectively), but not menopausal status.^16^

Using the SWAN cohort, 543 pre- and early perimenopausal women 42–52 years of age had annual measures of their weight, waist circumference, and body composition *via* bioelectrical impedance analysis.^38^ Weight increased 0.6% annually (*b* = 0.0056, 95% CI: 0.004–0.007) and waist circumference increased by 1% each year (*b* = 0.0065, 95% CI: 0.005–0.008). Based on the changes in FSH levels over the course of the 6 years these women were followed, no change in body composition was associated with menopausal status.^38^ Women continued to gain weight, fat mass, and waist circumference over the course of the study regardless of how many years since their FMP or if they were still premenopausal. However, the increase in waist circumference increased at a slower rate 1 year after FMP, which happened to align with the slower increased rate of FSH. This indicates an interaction of ovarian aging and hormonal transition with waist circumference but not with the observed linear increase in weight and fat mass.

The studies aforementioned showed strong correlations of age with adverse change in body composition; however, very few studies have been done to date showing a causal association. In a prospective study 63 early postmenopausal women 44–54 years of age were followed for 1 year.^39^ These women were split into two groups: women who used HRT and those who did not. There was an increase in weight and percent body fat in both groups, but no significant difference in the change between the groups. Since HRT was not able to prevent the weight gain seen in women during the menopausal transition, this study shows that midlife weight gain is likely due to age. Although a smaller sample, another study also found that women using HRT for 9 years or less (*n* = 21) did not have significant differences in weight gain or fat mass compared with postmenopausal women who did not use HRT.^40^

### Other factors that may influence weight changes during the menopause transition

Behavioral factors such as sleep patterns and physical activity levels may also affect weight changes and subsequent CVD risk in postmenopausal women.^41–44^ In a cross-sectional analysis of 507 women ages 20–79 years, better quality sleep was associated with a higher American Heart Association Life's Simple 7 (AHA LS7) score and thus stronger cardiovascular health.^41^ AHA LS7 score components are smoking, diet, physical activity, BMI, blood pressure, total cholesterol, and fasting glucose. The average age of the study sample was 37 years and roughly one-third were postmenopausal. In postmenopausal women, there was a significant interaction for sleep characteristics (*p* < 0.001) and a higher AHA LS7 score correlated to every 1-hour increase in sleep duration (β = 0.33, *p* = 0.013). This study found that lower AHA LS7 scores were associated with shorter sleep duration, poorer sleep quality, and greater insomnia severity in postmenopausal women but not premenopausal.

Further implicating the significance of AHAs LS7, the Women's Health Initiative Observational Study analyzed 27 variables' impact on weight gain in postmenopausal women (*n* = 612).^45^ Of the 27 variables investigated, weight gain ≥3% occurred significantly more often in women who had early menopause (<44 years), lower intake of dietary fiber, higher intake of fat and alcohol, and had/currently smoked.

A 5-year intervention study with premenopausal women 44–50 years of age further showed the beneficial impact of lifestyle changes on body composition in midlife women. The lifestyle intervention program included sessions on diet, cooking and physical activity, as well as support and motivation to ensure adherence to the program.^46^ After 4.5 years, 55% of the intervention group was at or below baseline weight, whereas only 26% of the controls maintained or lost weight. Maintained weight loss also correlated with improvement in LDL-C, triglycerides, and systolic blood pressure. The intervention group had the greatest adherence to the program's physical activity goal and consumed fewer total calories compared with the control group. Overall, this study showed that lifestyle intervention and education programs may prevent adverse changes in body composition during the peri- to postmenopausal period.

In another cross-sectional study, sleep quality *via* Pittsburgh Sleep Quality Index (PSQI) scores and body compositions were evaluated in 206 postmenopausal women who did not use HRT (mean age 61.4 years).^42^ Mean weight gain after menopause was 8.1 ± 12.8 kg. Around 57.8% of women had PSQI global scores ≥5, indicative of poor sleep quality and 47.6% had frequent waking throughout the night. Women who snored nearly every night also gained more weight during menopause than occasional snores. Although statistically insignificant, lower PSQI scores were associated with greater weight, BMI, and neck circumference values.

## Summary

**FIG. 1.**
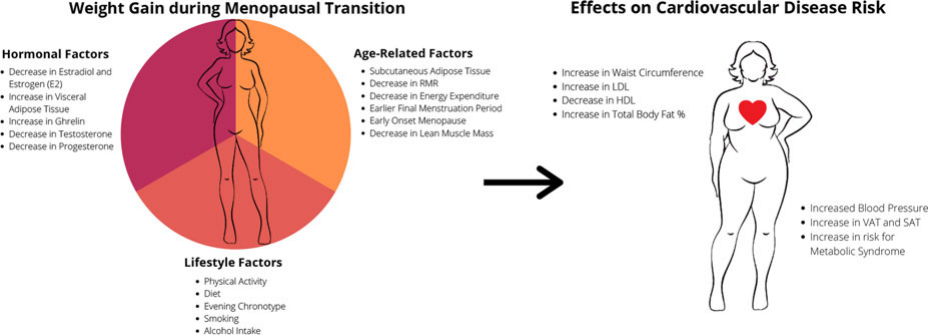
Factors associated with adverse weight changes during the menopausal transition and relation to CVD risk. CVD, cardiovascular disease.

In summary, physiological factors, including decreased estrogen concentration and changes in body fat distribution associated with the menopausal transition, as well as chronological aging, may make midlife women more prone to weight gain and subsequent obesity (Fig. 1). Increased weight accumulation during key lifetime transitions, such as the menopausal transition and the challenges associated with losing excess weight once it has been gained, can exacerbate cardiometabolic risk.

Overall, studies show that body weight gain often occurs during the menopausal transition in women ages 40–50 years, yet there are mixed results on whether these adverse weight changes occur *primarily* due to hormonal changes, aging, and/or behavioral/lifestyle factors. Studies have shown reduced weight gain in peri- and postmenopausal in women who use HRT while other studies have found no difference in weight status between menopausal women who use HRT and those who do not.

On the other hand, studies that focus on sex differences have indicated that weight gain around the time of the menopausal transition in women might be a result of aging as opposed to hormonal changes since it occurs in both men and women, when controlled for age. Behavioral factors such as poor diet, decreased physical activity, may also influence weight status in women undergoing menopause. In recent years, research has shown that sleep patterns, including short sleep duration and poor sleep quality also contribute to weight gain and these factors play roles in weight gain as women transverse menopausal transition in midlife.

Despite the identification of the menopausal transition as a key period of detrimental changes in women's adiposity levels and overall CVD risk, the number of studies that are inclusive of women undergoing this transition are extremely limited. Future research that includes longitudinal, comprehensive assessment of individual characteristics associated with adverse weight gain during the menopausal transition and how they contribute to overall cardiovascular health may provide necessary insights to inform educational efforts and strategies aimed at reducing the burden of CVD in women.

## Abbreviations Used

95% CI: 95% confidence interval
AHA LS7: American Heart Association Life's Simple 7
BMI: body mass index
CRP: C-reactive protein
CVD: cardiovascular disease
DXA: dual-energy X-ray absorptiometry
FMP: final menstrual period
FSH: follicle-stimulating hormone
HRT: hormonal replacement therapy
KNHANES: Korean National Health and Nutrition Examination Survey
LDL: low-density lipoprotein
MetS: metabolic syndrome
NHANES: National Health and Nutrition Examination Survey
PSQI: Pittsburgh Sleep Quality Index
SAT: subcutaneous adipose tissue
SWAN: Study of Women's Health across the Nation
tPA: tissue plasminogen activator antigen
VAT: visceral adipose tissue
YSM: years since menopause
